# Cochlear Glucocorticoid Receptor and Serum Corticosterone Expression in a Rodent Model of Noise-induced Hearing Loss: Comparison of Timing of Dexamethasone Administration

**DOI:** 10.1038/s41598-019-49133-w

**Published:** 2019-09-02

**Authors:** Seung-Hun Lee, Ah-Ra Lyu, Sun-Ae Shin, Seong-Hun Jeong, Sun-A Lee, Min Jung Park, Yong-Ho Park

**Affiliations:** 1 Department of Otolaryngology-Head and Neck Surgery, College of Medicine, Chungnam National University, Daejeon, Republic of Korea; 2 Department of Medical Science, College of Medicine, Chungnam National University, Daejeon, Republic of Korea; 3 Brain Research Institute, College of Medicine, Chungnam National University, Daejeon, Republic of Korea

**Keywords:** Cochlea, Trauma, Medical research, Molecular medicine

## Abstract

Glucocorticoid (GC) is a steroid hormone secreted from the adrenal cortex in response to stress, which acts by binding to cytoplasmic glucocorticoid receptors (GRs). Dexamethasone (DEX) is a synthetic GC exhibiting immunosuppressive effects in both human and rodent models of hearing loss. While clinical evidence has shown the effectiveness of DEX for treatment of various inner ear diseases, its mechanisms of action and the optimal timing of treatment are not well understood. In the present study, intergroup comparisons were conducted based on the time point of treatment with DEX: (1) pretreatment; (2) posttreatment; and (3) pre&post-noise. The pre&post DEX treatment group showed a significant improvement in threshold shift at 1 day post-noise exposure as compared to the TTS (transient threshold shift)-only group at 8 and 16 kHz. Both TTS and PTS (permanent threshold shift) significantly reduced cochlear GR mRNA expression and increased serum corticosterone and cochlear inflammatory cytokines. The pre&post DEX treatment group showed a significant decrease in serum corticosterone level as compared to other DEX treatment groups and TTS-treated group at 3 days after acoustic trauma. Our results suggest that the timing of DEX administration differentially modulates systemic steroid levels, GR expression and cochlear cytokine expression.

## Introduction

Glucocorticoids (GCs) are a class of steroid hormones secreted from the adrenal cortex in response to stress, which protect the organism against the negative effects of that stress^1–4^. They are among the most commonly prescribed drugs and are used for a wide range of medical conditions, including inner ear diseases, e.g., sudden idiopathic hearing loss^5^, acute noise-induced hearing loss^6^, and Ménière’s disease^7^. GCs exert powerful antiinflammatory effects by inhibiting several inflammatory mediators and increasing cochlear blood flow, to prevent hair cell damage caused by inflammation and ischemia in the inner ear^8–12^.

The actions of GCs are predominantly mediated through glucocorticoid receptors (GRs). GRs are ubiquitously expressed throughout the body, including the inner ear. In rodents, GRs are highly expressed in the stria vascularis, inner hair cells, outer hair cells (OHCs), and spiral ligament of the cochlea and cochlear nerve^13,14^. In humans, the highest level of GR expression is found in the spiral ligament, with the lowest level seen in the saccule^15^. Hearing function has been reported to be closely related to GR expression^11,15–19^.

Dexamethasone (DEX), a synthetic GC, is widely used clinically due to its antiinflammatory, antishock, and immunosuppressive properties. As shown in our previous studies and confirmed by other groups, systemic or local (intratympanic or intracochlear) application of DEX significantly rescues hearing loss^11,20–27^. Although reports in humans indicated the efficacy of DEX in intracochlear disorders, the mechanisms of action and timing of treatment have not been well established. Here, we investigated how noise trauma affects GR expression, corticosterone levels, and inflammatory responses. We also assessed the effects of the timing of DEX treatment for noise-induced hearing loss.

## Results

### Hair cell survival after noise exposure

**Figure 1:**
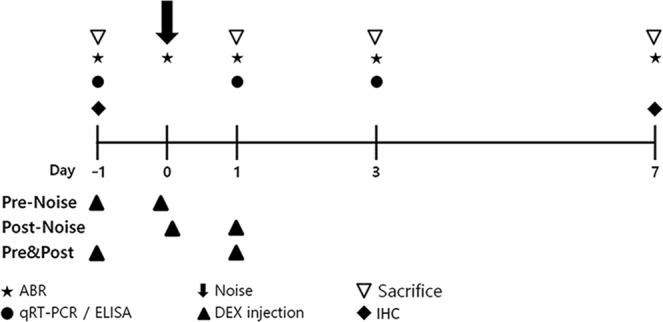
Schematic of the experimental design. Auditory brainstem response (ABR) thresholds were measured at four time points: prior to, immediately after, and 1, 3 and 7 days after noise exposure. Two dexamethasone (DEX) injections were given to animals in three treatment groups: (1) pre-noise group, injections at 1 day before and immediately prior to noise exposure; (2) post-noise group, injections immediately after and 1 day after noise exposure; and (3) pre&post-noise group, injections at 1 day before and 1 day after noise exposure. Serum and/or tissue lysates were collected prior to and at 1, 3, and 7 days after noise exposure for biochemical analyses, including quantitative real-time polymerase chain reaction (qRT-PCR) and enzyme-linked immunosorbent assay (ELISA).

**Figure 2:**
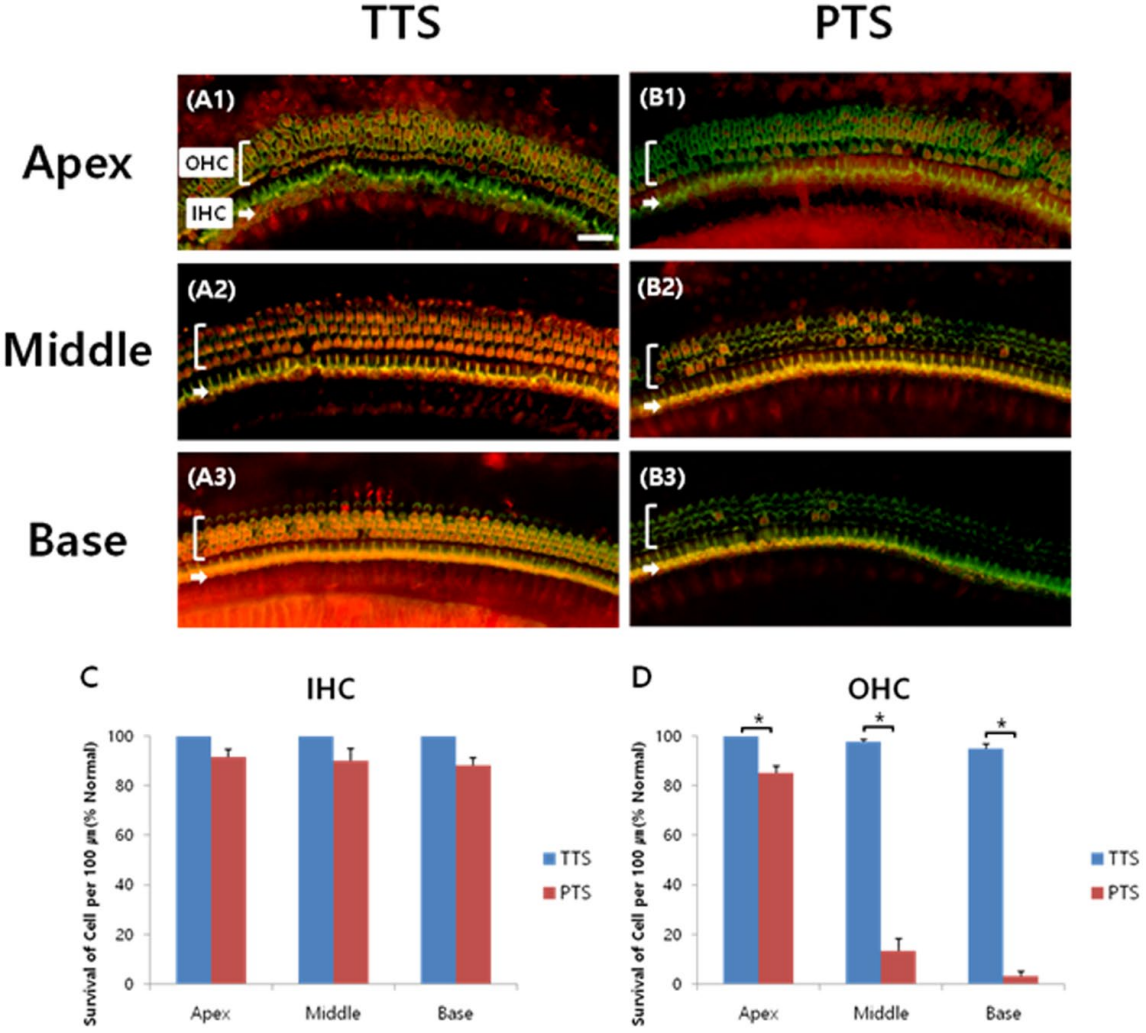
Whole-mount preparations of the auditory epithelium in the TTS (A1–A3) and PTS (B1–B3) groups. Tissues were stained for myosin VIIa (red) to visualize the hair cells and then photographed using epifluorescence. (**A**,**B**) Outer hair cells were more noticeably destroyed on the middle and basal turns of the cochlea in the PTS group (B1–B3) compared to the TTS group (A1–A3). Scale bar: 30 µm. (**C**,**D**) Quantitative analysis of hair cell survival on IHCs (**C**) and OHCs (**D**): apex, middle, and basal turns. TTS, transient threshold shift; PTS, permanent threshold shift; A1 and B1, apical turn; A2 and B2, middle turn; A3 and B3, basal turn; OHC, outer hair cell; IHC, inner hair cell. All graphs represent mean ± S.E.M. n = 3 each group. *p < 0.05. Unpaired T-test.

Healthy CBA/J male mice were randomly assigned to two groups according to the noise exposure level: a transient threshold shift (TTS) group and a permanent threshold shift (PTS) group. To examine whether TTS or PTS causes hair cell loss/survival in the cochlea, whole-mount preparations of the auditory epithelium were stained with antibodies against myosin VIIa (red, hair cells) and phalloidin-FITC (green, F-actin) at 7 days after noise exposure as the schedule of the experiments is shown in Fig. 1. OHCs were more noticeably destroyed in the middle and basal turns of the cochlea in the PTS group (Fig. 2B1–B3) compared to the TTS group (Fig. 2A1–A3). Quantitative analysis of hair cell survival showed that PTS induced a significantly lower level of OHC survival (apex, middle, and basal turns, p < 0.05) compared to TTS (Fig. 2D). Loss of OHC was more prominent in the basal and middle turns compared to the apex turn in the PTS group (Fig. 2B2,B3,D). There was no significant difference in inner hair cell loss between TTS and PTS (Fig. 2C).

### Expression of glucocorticoid receptor in the normal cochlea

**Figure 3:**
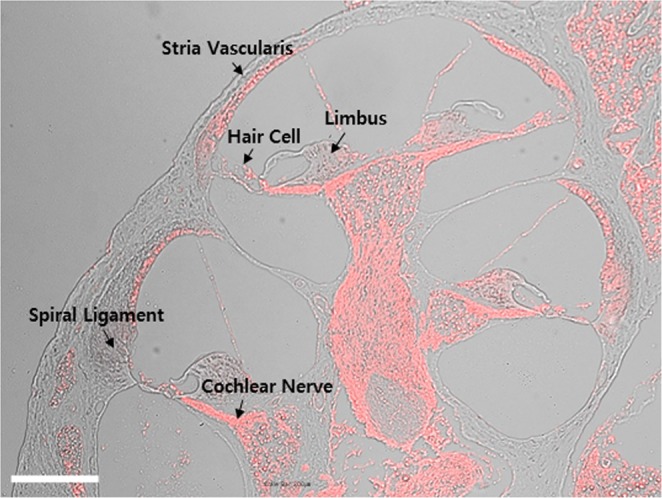
Localization of glucocorticoid receptors (GRs) in the mouse cochlea. Immunohistochemical analysis indicated that GRs (red) were widely expressed in the cochlea, including the stria vascularis, hair cells, limbus, spiral ligament, and cochlear nerve.

GCs represent the only clinically proven treatment for various otological disorders and are known to exert their effects through GRs. Here, we determined which regions in the cochlea express GR. Immunohistochemistry for GR (labeled in red; Fig. 3) revealed wide expression of GR in the cochlea, specifically in the stria vascularis, hair cells, limbus, spiral ligament, and cochlear nerve (Fig. 3). The immunostaining results indicated that GR are significantly expressed in the whole cochlea under normal conditions.

### Changes in GR mRNA expression

**Figure 4:**
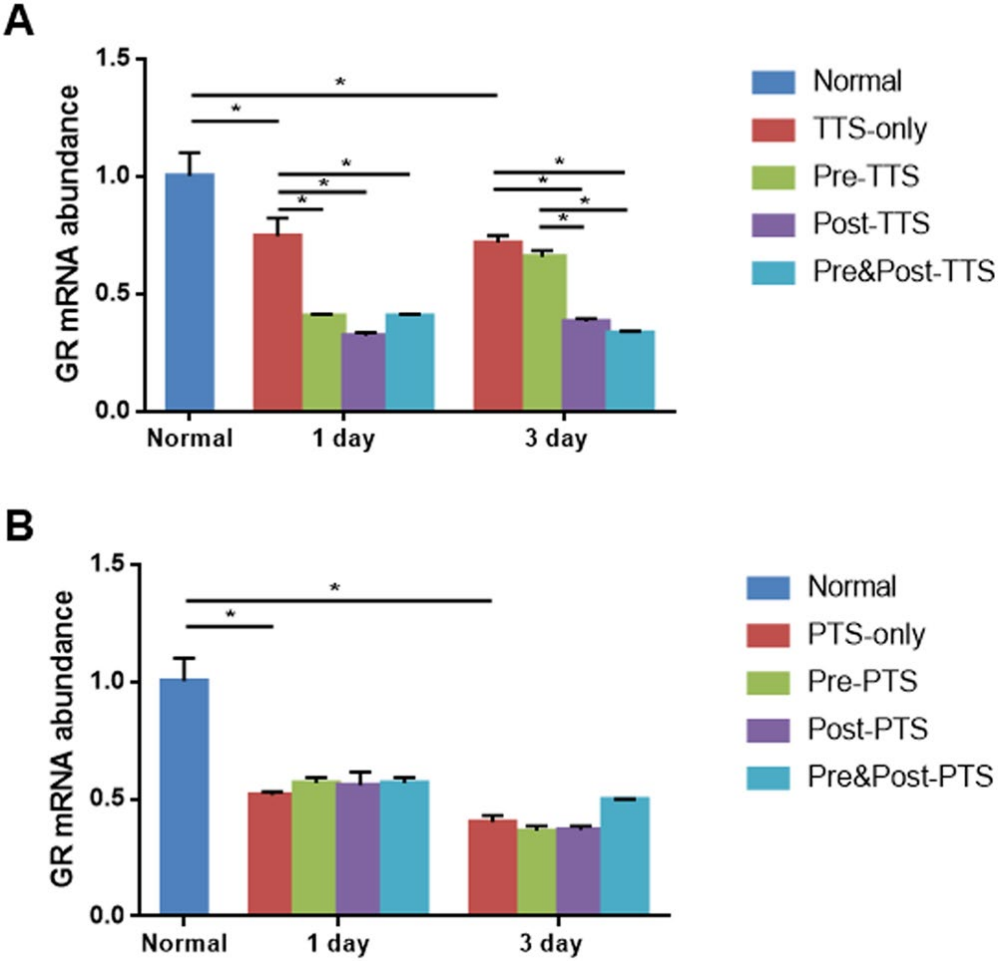
GR mRNA expression following noise exposure. (**A**) In the TTS group, GR mRNA expression was significantly decreased at 1 day after noise exposure compared to the untreated group. All three DEX-treated groups (pre-noise, post-noise, and pre&post-noise) showed significantly decreased GR mRNA expression at 1 day after noise treatment, and the change was maintained in the post-noise and pre&post-noise treatment groups at 3 days after noise exposure. (**B**) In the PTS treatment group, GR mRNA expression was significantly decreased after noise exposure, regardless of DEX treatment, at both days 1 and 3. All graphs represent mean ± S.E.M. n = 3 each group, total 54 mice were used. One-way ANOVA and Tukey’s multiple comparisons test. *p < 0.05.

We next evaluated the impact of GR expression in the cochlea after noise exposure by treatment with the synthetic GC, DEX. Eight-week-old CBA/J male mice were randomly assigned to one of five groups: 1) normal; 2) noise exposure (TTS or PTS); and three DEX groups. Two DEX injections were administered at different time points: (1) pre-noise group (Pre-TTS/PTS), injections at 1 day before and immediately prior to noise exposure; (2) post-noise group (Post-TTS/PTS), injections immediately after and 1 day after noise exposure; and (3) pre&post-noise group (Pre&Post-TTS/PTS), injections 1 day before and 1 day after noise exposure (Fig. 1). Cochlear samples were collected at 1 and 3 days after noise exposure and analyzed for GR mRNA expression. As shown in Fig. 4A, TTS induced a significant decrease in GR mRNA expression at 1 day (normal vs. 1 day TTS-only, P = 0.0002) and 3 days (normal vs. 1 day TTS-only, P < 0.0001) after noise exposure. All DEX-administered subgroups (pre-noise, post-noise, and pre&post-noise) showed a significant decrease in GR expression in comparison to normal mice, as well as TTS-only animals (Tukey’s multiple comparison test, P < 0.05) at 1 day after noise exposure. The lower level of GR expression was maintained at 3 days after noise exposure in post- and pre&post-DEX mice compared to the TTS-only group (Fig. 4A). The PTS group also showed a significant decrease in GR expression compared to normal mice at both 1 day and 3 days after noise exposure. Interestingly, the DEX-administered subgroups (pre-noise, post-noise, and pre&post-noise) showed significant decreases in GR expression compared to normal animals; however, no significant changes were found in the DEX-administered subgroups compared to the PTS-only group (Fig. 4B), suggesting that GR expression induced by PTS decreased further than that induced by TTS, and thus diminished differences between PTS-only and PTS + DEX (all three subgroups). These data indicated that GR mRNA expression is significantly affected by noise exposure and DEX administration and that post and pre + post DEX treatments are comparable in GC receptor expression level.

### Auditory brainstem response (ABR) threshold shifts

**Figure 5:**
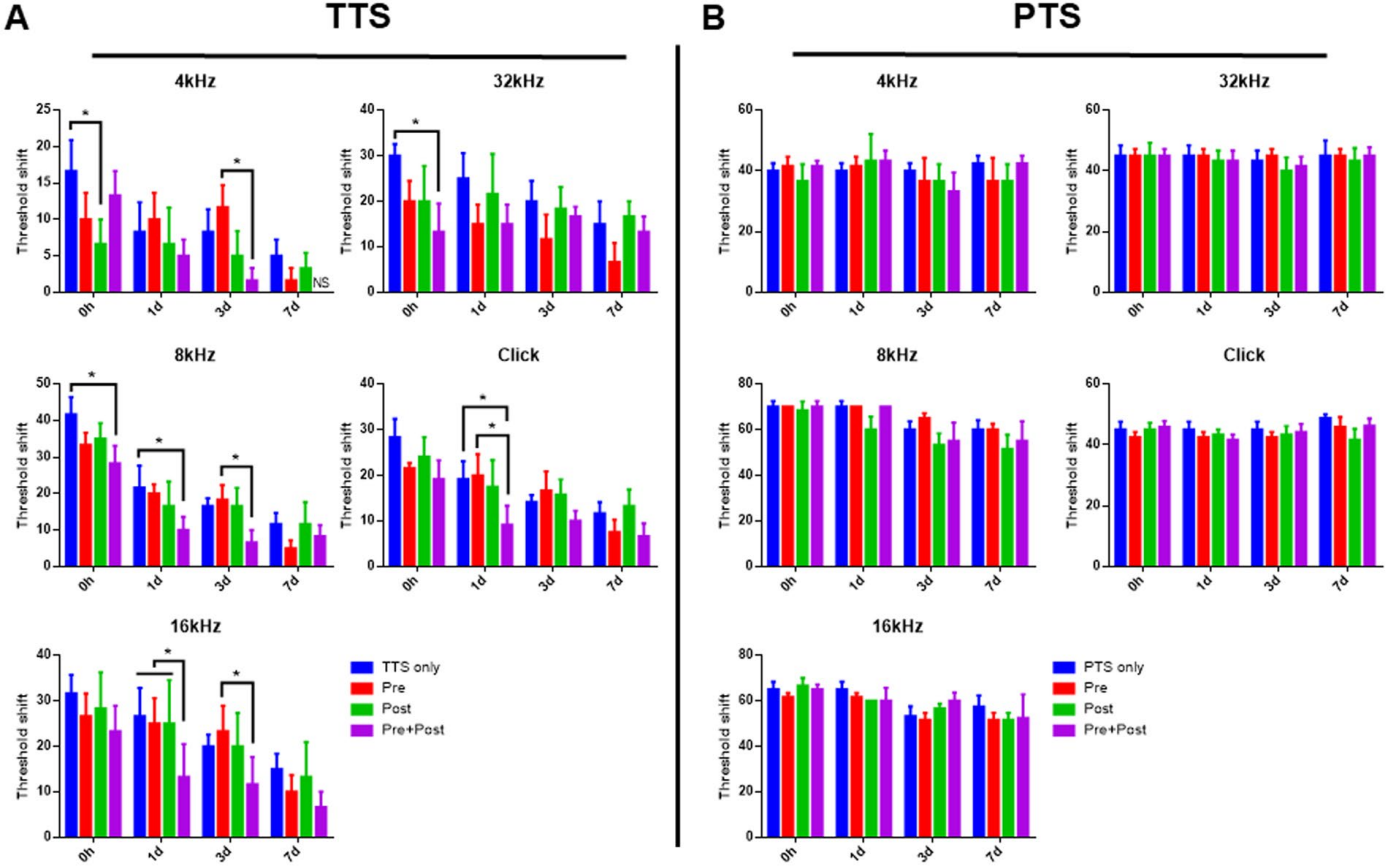
ABR threshold shifts with or without DEX administration. ABR thresholds were measured at five time points: prior to, immediately after, and 1, 3 and 7 days after noise exposure. (**A**) In the TTS group, increased ABR threshold shifts were observed in all groups after noise exposure and gradually decreased over time. ABR threshold shifts were significantly reduced in the pre&post DEX group compared to the TTS-only group at some frequencies and click. (**B**) PTS induced prolonged ABR threshold shifts at all time points until 7 days after noise exposure. There were no significant differences among treatment groups. All graphs represent mean ± S.E.M. Two-way repeated measures ANOVA and Tukey’s multiple comparisons test (n = 6 each group, total 48 mice were used). *p < 0.05.

To evaluate how noise exposure changes the hearing threshold in different groups, the ABR thresholds at 4, 8, 16, and 32 kHz, and click sounds were measured at four time points as indicated in Fig. 1: before noise exposure (baseline); immediately after; 1, 3, and 7 days following noise exposure. As shown in Fig. 5A, the ABR threshold shift significantly increased immediately after noise exposure and showed a declining pattern over the next 7 days, indicating that the animals recovered from TTS over time. Pre&post DEX treatment significantly improved threshold shift as compared to TTS-only at 8 kHz (Two-way Repeated Measures ANOVA; Tukey’s multiple comparisons test; n = 6 each group; 0 day, p = 0.0088; and 1 day, p = 0.0266), 16 kHz (1 day, p = 0.0056), 32 kHz (0 hr, p = 0.0212), and click (1 day, p = 0.0293). Moreover, pre&post treatment was significantly different from the pre treatment at 4 kHz (3 day, p = 0.0422), 8 kHz (3 day, p = 0.0266), and 16 kHz (1 day, p = 0.0185). Threshold shift at 16 kHz (Two-way Repeated Measures ANOVA; Tukey’s multiple comparisons test; n = 6 each group; main effect time, F _(3, 15)_ = 9.441, p = 0.0009) also revealed that pre&post DEX significantly improved hearing loss at 1 day post noise exposure as compared to all other groups (pre&post vs. pre, p = 0.0185; pre&post vs. post, p = 0.019). No differences in ABR threshold shifts were observed in DEX treatment groups compared to the PTS-only group (Fig. 5B). Collectively, these data support that the timing of DEX administration differentially effects functional recovery after noise trauma.

### Circulating corticosterone level with noise exposure and DEX administration

**Figure 6:**
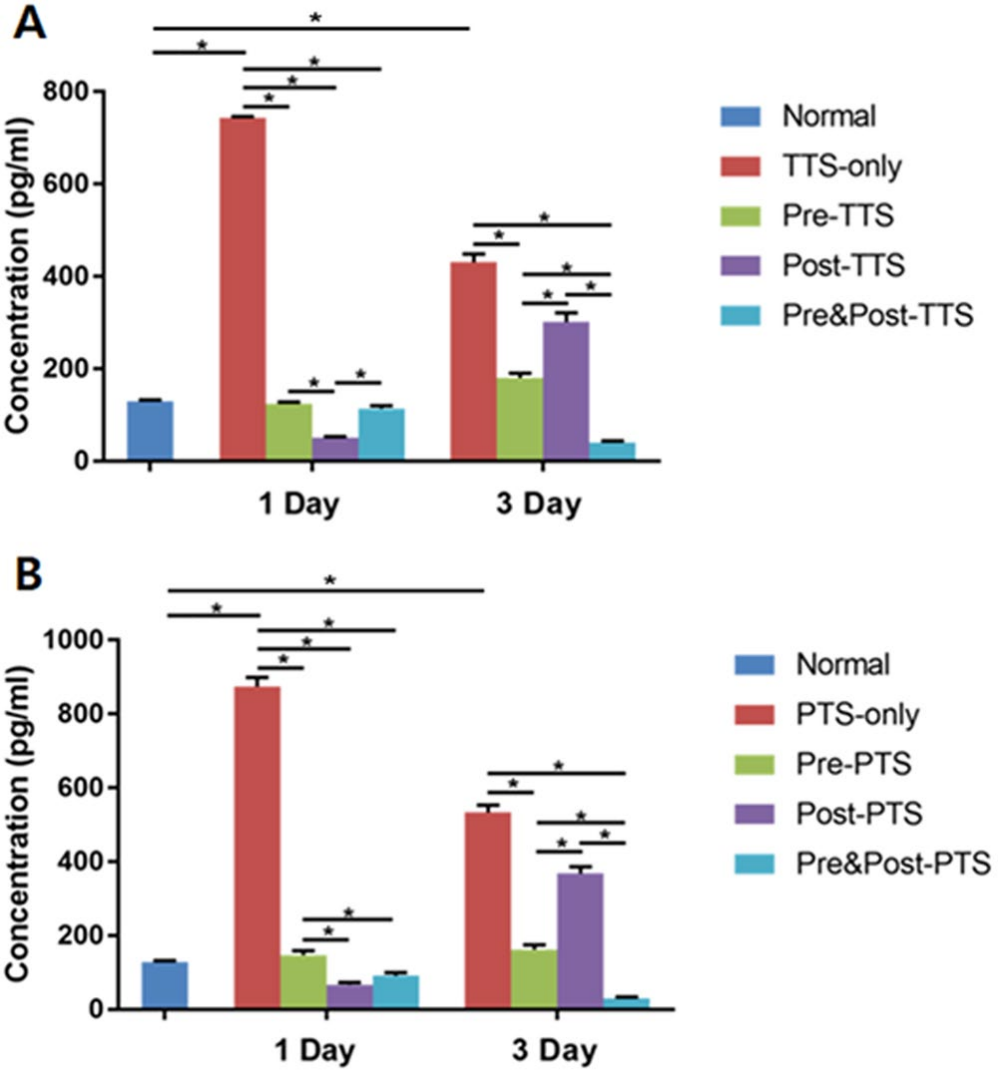
Serum corticosterone levels at 1 and 3 days after noise exposure. Noise exposure (both TTS and PTS) significantly increased the circulating corticosterone level at early (1 day) and late (3 days) acute phases after noise exposure. DEX administration maintained a low circulating level of corticosterone after noise trauma. Interestingly, the post-DEX group showed significantly lower levels of corticosterone compared to the pre-DEX and pre&post-DEX groups in the early acute phase (1 day). At the late acute phase (3 days), post-DEX showed significantly increased serum corticosterone levels in comparison to the other DEX treatment groups. All graphs represent mean ± S.E.M. One-way ANOVA and Tukey’s multiple comparisons test (n = 3 each group, total 54 mice were used). *p < 0.05.

To test circulating levels of corticosterone, serum samples were collected from mice before (normal) or after (1 day and 3 days) noise exposure and assayed for corticosterone. The corticosterone levels are shown in Fig. 6. TTS induced a significant increase in serum corticosterone level compared to normal mice at 1 and 3 days after noise exposure (Fig. 6A). DEX subgroups did not show a significant increase in the corticosterone level compared to normal mice at 1 day after noise exposure, indicating that DEX treatments prevented the increase in corticosterone level after noise trauma (Fig. 6A). The post-DEX group (post-TTS) showed a similar corticosterone level to the TTX-only group at 3 days following noise exposure, whereas the corticosterone levels in the other DEX groups (pre- and pre&post-TTS) remained decreased compared to the TTS-only group at 3 days after noise trauma (Fig. 6A). PTS also caused significant increases in circulating corticosterone levels compared to normal animals at 1 and 3 days after noise exposure (Fig. 6B). The DEX subgroups showed a similar pattern in terms of PTS as observed in the TTS experiments (Fig. 6A). These observations suggested that serum corticosterone levels are significantly affected by noise exposure and DEX administration.

### Changes in inflammatory cytokines

**Figure 7:**
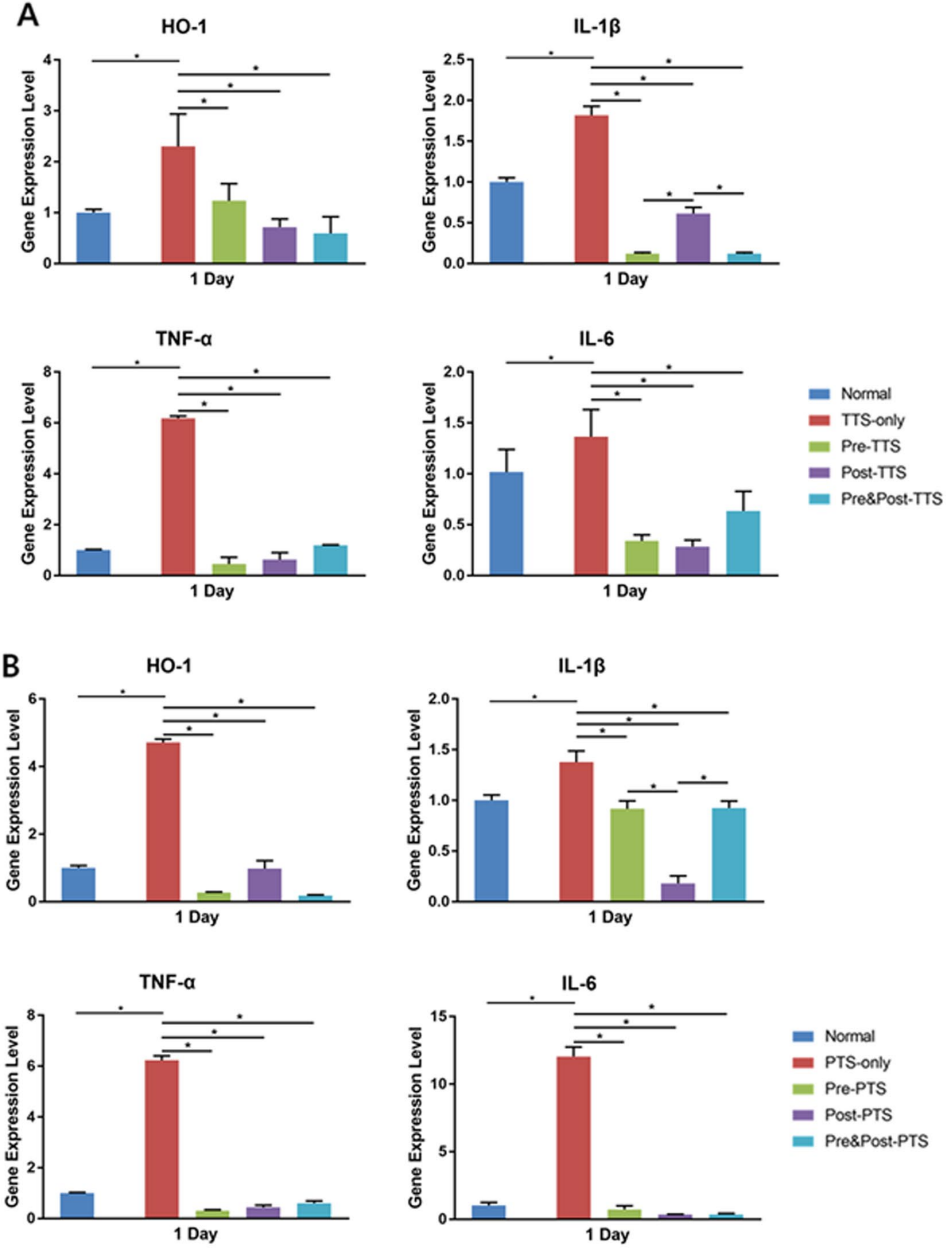
Inflammatory cytokine expression in the cochlea. Both TTS (7A) and PTS (7B) significantly increased expression levels of heme oxygenase-1(HO-1), interleukin-1β (IL-1β), tumor necrosis factor-α (TNF-α), and interleukin-6 (IL-6) in the cochlea compared to non-treated animals. IL-1β was significantly decreased in the pre- and pre&post-TTS groups compared to the post-TTS group, and in the post-PTS group compared to the pre- and pre&post-PTS groups. All graphs represent mean ± S.E.M. *One-way ANOVA and Tukey’s multiple comparisons test (n = 3 each group, total 30 mice were used). *p < 0.05.

To test cytokine levels in the cochlea, we collected tissue samples from the five groups described above at 1 day after noise trauma and performed quantitative real-time polymerase chain reaction (qRT-PCR) assays for heme oxygenase-1 (HO-1), interleukin-1β (IL-1β), tumor necrosis factor-α (TNF-α), and interleukin-6 (IL-6). Figure 7 shows that the expression levels of HO-1 and the proinflammatory cytokines, IL-1β, TNF-α, and IL-6, were significantly increased in TTS- and PTS-treated groups compared to normal animals. DEX administration significantly decreased the expression levels of the cytokines and HO-1 compared to the TTS- and PTS-only groups. No significant differences were found among the three DEX treatment groups. These observations suggested that exposure to noise induces a local cochlear inflammatory response, which can be ameliorated by DEX administration.

## Discussion

The results of this study showed that noise trauma decreased cochlear GR expression (Fig. 4), and increased both serum corticosterone level (Fig. 6) and cochlear cytokine expression (Fig. 7). Treatment with the synthetic GC, DEX, further decreased the GR mRNA level in the cochlea (Fig. 4), and prevented increases in serum corticosterone and cytokine levels (Figs 6 and 7). This study also provided insight into the potential mechanism underlying the effectiveness of DEX for rescuing noise-induced hearing loss. Threshold shift at 16 kHz revealed that pre&post DEX significantly improved hearing loss at 1d post noise trauma as compared to all other groups (Fig. 5).

GC is widely used for the treatment of inner ear disease, exerting its effects by binding to its receptor, GR^2^. Therefore, the expression of GR mediates the effects of GC^28^. GRs are highly expressed throughout the inner ear, including the stria vascularis, inner hair cells, OHCs, and spiral ligament of the cochlea and cochlear nerve^13,14^. The GR mRNA expression level was reported to be significantly decreased following acoustic trauma in the cochlea^29^ as well as in the organ of Corti^30^. Terunuma *et al*. reported that GR mRNA expression was significantly decreased following acoustic trauma^29^. Mori *et al*. reported that compound action potentials (CAPs) at a threshold of 5–8 kHz were significantly elevated when the GR antagonist, mifepristone, was administered following exposure to noise with a sound pressure level (SPL) of 120 dB^31^. The ABR threshold shift was found to be significantly decreased by pretreatment with the corticosteroid, methylprednisolone^32^. The GR antagonist, RU486, and the GC synthesis inhibitor, metyrapone, significantly increased the ABR threshold shift^32,33^. In the present study, we confirmed that noise trauma, including TTS and PTS, significantly decreased GR mRNA expression in the cochlea (Fig. 4).

The results shown in Fig. 4 raise the question of how TTS and PTS differentially affect local cochlear GR mRNA expression. GR expression was further diminished by PTS compared to TTS at 1 day (0.5185 ± 0.0074 vs. 0.7468 ± 0.0441, *P* = 0.0070, unpaired *t* test) and 3 days (0.4035 ± 0.0154 vs. 0.7180 ± 0.0177, *P* = 0.0002, unpaired *t* test), suggesting that GR expression may be inversely correlated to the degree of stress. The expression levels of the GR isoform, GRα, were shown to be negatively correlated with the number/degree of stressful events experienced in a clinical study of posttraumatic stress disorder (PTSD)^34^. Although DEX partially rescued TTS-induced hearing impairment (Fig. 5A), it was ineffective in the PTS group (Fig. 5B). These observations suggested that the dose and/or timing of DEX administration were insufficient to rescue the permanent cell damage (as shown in Fig. 2) induced by intense acoustic trauma (PTS).

The noise trauma increased the endogenous circulating corticosterone level (Fig. 6), with a subsequent decrease in cochlear GR mRNA expression (Fig. 4). The magnitude of hearing loss induced by noise trauma was decreased by pre&post treatment with the GR agonist, DEX (Fig. 5), which was accompanied by diminished cochlear GR mRNA expression (Fig. 4). Tahera *et al*. reported that treatment with the GR antagonist, RU486, and the GC synthesis inhibitor, metyrapone, prior to acoustic trauma increased GR mRNA expression in the cochlea^33^. In contrast, Helling’s group reported that pretreatment with the GR agonist, DEX, significantly increased GR expression compared to a noise exposure group in a different region of the cochlea, the spiral ligament^25^. Further studies to investigate how acoustic trauma and/or DEX treatment affect GR expression in specific subregions of the cochlea are required.

Acoustic trauma is one of the stressors that raise circulating corticosterone level^35^ by stimulating the hypothalamic-pituitary-adrenal axis^33^. In this study, serum corticosterone levels were increased in both TTS and PTS groups after noise exposure (Fig. 6). The serum corticosterone level was as low as in normal mice at 1 day in all DEX treatment groups, and this low level was maintained until 3 days only in the pre&post DEX treatment group (Fig. 6), consistent with the improved hearing function in the same group (Fig. 5). It is possible that exogenous DEX administration plays a role in maintaining homeostasis by acting as an endogenous source of corticosterone in the cochlea. The decrease in GR mRNA level may be due to feedback inhibition by increased endogenous stress hormone, corticosterone after acoustic stress as shown in Fig. 6. We speculate that the increase may be an acute response after noise stress and maintained at a high level for at least 3 days after the trauma. The small nitric oxide (NO) molecule could be responsible for the increase in GR mRNA expression after noise stress. Noise exposure significantly increases the NO production in the cochlea, which may degrade proteins by ubiquitination and/or proteasomal activity of the cytochrome P450^36^. Another potential mechanism may be differences in the phosphorylation status of the GR mRNA. The mouse GR contains eight phosphorylation sites, so that the phosphorylation status of GR may pre-determine the GR protein turnover and local GR degradation after stress^37–40^.

While we found a diminished GR expression after noise trauma and a further downregulation of GR in DEX treatment groups (Fig. 4) from the whole cochlear extracts, Heinrich *et al*. reported DEX treatment restored GR intensity as compared to noise trauma per se in Guinea pig. Others have reported contradicting outcomes by showing that DEX down-regulates GR mRNA expression in rat hepatoma culture (HTC) cells^41^. They presented that glucocorticoid-treatment for 24–48 hr resulted in a down-regulation of cellular GR mRNA levels in both HTC cells and in rat liver *in vivo*^41^. One of the important observations was the transient downregulation of GR, since the GR mRNA level was restored after 72 hr^41^. More interestingly, an initial increase of GR mRNA was observed before the down-regulation occurred. These reports indicate that GR mRNA expression is affected in a time-sensitive manner after DEX treatment, and its response may be variable depending on tissue- and/or cell-types. Steroid hormone receptors are well known to function as transcription factors mediating the biological effects of steroids by regulating gene expression. However, it is also reported that the receptors are regulated both by transcriptional and posttranslational mechanisms^42^. This implies that measuring the GR protein levels along with mRNA expression may be important to access DEX-induced GR alterations. Therefore, in-depth studies on GC and GR mechanisms are further needed.

Cytokines, including IL-1β, TNF-α, and IL-6, were maintained at low levels after DEX administration compared to the noise exposure-only group (Fig. 7). These proinflammatory cytokines have been suggested to cause brain damage similar to free radicals^43^. Several studies reported the presence of inflammatory cells and cytokines in the cochlea following acoustic trauma^44,45^ with a significantly increased ABR threshold shift^33^.

A key question is how GC and GR signaling modulates inflammatory cytokines. GR signaling is known to be mediated by two pathways: a classical genomic (DNA binding-dependent) pathway and a non-genomic (DNA binding-independent) pathway. The classical genomic pathway modulates the expression of target genes by binding to specific DNA sequences within the nucleus after GC binds to the GR^6,46,47^. Some of the GRs associate with transcription factors, such as AP-1 and NFκB (essential molecules in upregulation of the inflammatory response), which is the pathway by which GCs exert the majority of their antiinflammatory effects^48^. The non-genomic (DNA binding-independent) pathway can modulate gene expression without binding to hormone response elements (HREs), and is also considered to underlie a number of immunosuppressive and antiinflammatory actions^2,4,33,49,50^.

Previous studies have used various doses of DEX. For instance, Weichhart *et al*. utilized DEX intraperitoneally at 600 μg (∼30 mg/kg) to study the immune suppressive effects of DEX after LPS-induced inflammation in a mouse model^51^. Tuckermann *et al*. injected DEX intraperitoneally at a dose of 25 μg and subsequently continued with 20 mg/L dexamethasone (drinking water) in C57BL/6 and Balb/c mice^52^. Sadikot *et al*. used DEX in a range of 0.3 ug/g to 10 ug/g in transgenic reporter mice (HLL mice) to investigate DEX treatment on NF-kappa B activation^53^. These reports support that our usage of DEX concentration, 20 ug/g (=20 mg/kg), is not far from the doses generally utilized in pre-clinical studies. However, regardless the conventional-dose of DEX in animal studies, our DEX dose far exceeds the dose used in humans. Therefore, further preclinical studies with DEX concentrations that can be reasonably translated to human dose seem necessary.

In summary, the results of the present study indicated that serum corticosterone level and GR expression are important for maintenance of cochlear homeostasis. In addition, DEX administration may be recommended before and after acoustic trauma, especially in soldiers before leaving for a battlefield, which may minimize noise-induced hearing loss by regulating GR mRNA and immune responses.

## Methods

### Animals

All animal experiments were approved by Chungnam National University, Institutional Animal Care and Use Committee (CNU00936). CBA/J male mice, aged 8 weeks, weighing 25–30 g, were confirmed to have normal hearing prior to noise exposure, were used in this study. Healthy mice were randomly divided into two groups according to the noise exposure level: a transient threshold shift (TTS) group and a permanent threshold shift (PTS) group. Each group was further divided into four subgroups according to the timing of DEX administration: noise exposure only, pre-noise, post-noise, and pre&post-noise. The experimental animals were used for time point studies on days 1, 3, and 7 following exposure. To avoid any impact of circadian rhythm on hormone levels, serum samples were collected at the same time of day (between 12:00 and 14:00). The schedule of the experiments is shown schematically in Fig. 1.

### Noise exposure

In the TTS groups, animals were exposed to free-field broadband noise (250 Hz–8 kHz) for 20 minutes at an intensity of 116 decibels (dB) SPL following previously-published protocols^20^. In the PTS groups, free-field broadband noise (2–8 kHz) was applied for 2 hours at an intensity of 116 dB SPL in an acoustically insulated reverberation chamber as described previously^20,54^. The noise signals were routed through a computer and an amplifier (INTER-M R300 Plus power amplifier; Canford Audio PLC, Washington, UK) to a loud speaker (ElectroVoice DH1A-WP; Sonic Electronix Inc., Sylmar, CA). The noise level was measured using a sound level meter (B&K type 2250; Brüel & Kjaer, Naerum, Denmark), sound calibrator (B&K type 4231; Brüel & Kjaer), and condenser microphone (B&K type 4189; Brüel & Kjaer).

### Dexamethasone injection

DEX (5 mg/mL dexamethasone sodium phosphate; Huons, Sungnam, Korea) was injected intraperitoneally at a dose of 20 μg/g. The pre-noise subgroups were injected with DEX at 1 day before and immediately before noise exposure. The post-noise subgroups were administered DEX immediately after and 1 day after noise exposure, and the pre&post-noise subgroups were administered DEX at 1 day before and 1 day after noise exposure. Noise exposure-only subgroups did not receive DEX injection.

### Auditory brainstem response

ABR thresholds at frequencies between 4 and 32 kHz, and click sounds, were obtained separately from both ears as described previously^20^. ABRs were recorded prior to noise exposure, immediately after, and 1, 3 and 7 days after noise exposure. The TDT System-3 (Tucker Davis Technologies, Gainesville, FL) hardware and software were used to obtain the ABRs. The stimuli were computer-generated tone pips.

The animals were anesthetized with intramuscular injection of zolazepam HCl 40 mg/kg (Zoletil, Virbac Animal Health, Carros, France) and xylazine 10 mg/kg (Rompun, Bayer Animal Health, Monheim, Germany)^20^. Subcutaneous needle electrodes were placed around the skull vertex and both infraauricular areas. Tone bursts, with a duration of 4 ms and rise-fall time of 1 ms at frequencies of 4, 8, 16, 32 kHz, were used, in addition to clicks. The sound intensity was varied in 10-dB increments for the tone burst sounds and in 5-dB increments for the click and tone burst sounds close to the threshold. The contralateral ear was not masked because the stimuli were transmitted through a sealed earphone. The waveforms were analyzed using a custom program (BioSig RP, ver. 4.4.1; Tucker Davis Technologies) with the researcher blinded to the treatment group. Threshold was defined as the lowest stimulus intensity to evoke a wave III response >0.2 μV.

### Quantitative real-time polymerase chain reaction

Animals were sacrificed at either 1 or 3 days after the surgical procedures and qRT-PCR was performed to evaluate the expression of GR and degree of inflammation. HO-1, IL-1β, TNF-α, and IL-6 were measured as indicators of inflammatory response^20,54^.

Dissected cochleae were ground in 1 ml of TRIzol reagent (Invitrogen, Carlsbad, CA), and 200 μl of chloroform was added followed by centrifugation at 13,000 rpm for 15 minutes. About 450 μl of supernatant was transferred to a fresh tube and an equal volume of isopropanol was added, shaken for 5 minutes, and centrifuged at 13,000 rpm for 15 minutes. The resulting pellet was resuspended in 1 ml of 80% ethanol in DEPC-treated water and centrifuged at 13,000 rpm for 15 minutes. The same procedure was performed one more time and the pellet was then washed repeatedly with 100% ethanol. RNA was dissolved in 20 μl of RNase-free water. The purified RNA was quantified using a Nanodrop instrument (NanoDrop Technologies Inc., Wilmington, DE) by measuring the absorbance at 260 nm. A total of 13 μl of RNA (2 μg each) with oligo-dT primer and DEPC-treated water was pre-denatured for 10 minutes at 65 °C; 4 μl of 5 × reaction buffer, 2 μl of dNTP, 0.5 μl of RNase inhibitor, and 0.5 μl of reverse transcriptase were added and reverse-transcribed for 1 hour at 50 °C and 5 minutes at 85 °C with a cDNA Synthesis Kit (Roche, Indianapolis, IN). Real-time reverse transcription was performed according to the manufacturer’s protocol with SYBR Green (Invitrogen, Grand Island, NY). Comparative quantification of GR, HO-1, IL-1β, TNF-α, and IL-6 mRNA was performed using the cycle threshold method. qRT-PCR was performed three times for each sample. Details of the primers used in PCR to detect GR, HO-1, IL-1β, TNF-α, and IL-6 are presented in Table 1.

**Table 1:** Primer sequences used in this study for quantitative RT-PCR.

Primer name		Sequence (5′-3′)
GAPDH	Forward	5′-TGTGTCCGTCGTGGATCTGA-3′
	Reverse	5′-CCTGCTTCACCACCTTCTTGAT-3′
GR	Forward	5′-CCCAAGAGTTCAACACCTGC-3′
	Reverse	5′-AAACTCCTTCTCTGTCGGGG-3′
HO-1	Forward	5′-CCCACCAAGTTCAAACAGTCT-3′
	Reverse	5′-AGGAAGGGGGTCTTAGCCTC-3′
IL-1β	Reverse	5′-TCTTTGAAGTTGACGGACCC-3′
	Reverse	5′-TGAGTGATACTGCCTGCCTG-3′
TNF-α	Forward	5′-CTGAGGTCAATCTGCCCAAGTAC-3′
	Reverse	5′-CTTCACAGAGCAATGACTCCAAAG-3′
IL-6	Forward	5′-TCGTGGAAATGAGAAAAGAGTTG-3′
	Reverse	5′-AGTGCATCATCGTTGTTCATACA-3′

### Enzyme-linked immunosorbent assay

After sacrificing the animals, blood samples were collected in heparinized tubes at the same time of day to minimize the circadian fluctuation of corticosterone level (between 12:00 and 14:00). Plasma was separated by spinning at 3,000 rpm at room temperature, and was then immediately stored at −20 °C until corticosterone assay. The serum corticosterone level was determined using an ELISA kit (Corticosterone ELISA kit, ADI-900-097; Enzo Life Sciences, Farmingdale, NY) with a sensitivity of 5 pg/ml.

### Tissue preparation and immunohistochemistry

The animals were sacrificed before (Fig. 3) or 7 days after noise exposure (Fig. 2). Cochlear tissues were obtained to localize glucocorticoid receptors (Fig. 3) and assess the survival of hair cells and nerve fibers (Fig. 2). Tissues were fixed in 4% paraformaldehyde in phosphate-buffered saline (PBS) for 1 hour at room temperature. After removal of the cochlear bony walls and lateral wall tissues, the remaining cochlear tissues were prepared for immunostaining. Tissues were permeated with 0.3% Triton X-100 (Sigma-Aldrich, St. Louis, MO) for 10 minutes, blocked in 5% normal goat serum (Vector Laboratories, Burlingame, CA) for 30 minutes, and then incubated with mouse anti-GR primary antibody (Santa Cruz Biotechnology, Dallas, TX) - Alexa 594 (Invitrogen-Molecular Probes, Eugene, OR) or rabbit anti-myosin VIIa primary antibody (Proteus BioSciences, Ramona, CA) - Alexa Fluor 488 Phalloidin (A12379; Invitrogen-Molecular Probes, Eugene, OR) at a concentration of 1:200 in blocking solution overnight at 4 °C. After rinsing in PBS for 10 minutes, the tissues were incubated with the AlexaFluor 594 goat anti-rabbit secondary antibody (Molecular Probes) at a concentration of 1:200 in PBS for 30 minutes. After rinsing in PBS for 10 minutes, specimens were further dissected to separate individual cochlear turns, and mounted on glass slides using Crystalmount (Biomeda, Foster City, CA). The specimens were observed under an epifluorescence microscope (Zeiss Axio Scope A1; Zeiss, Oberkochen, Germany) with a digital camera. The timelines for all experiments are shown in Fig. 1.

### Image processing and statistical analysis

Adjustment of image contrast, superimposition of images, and colorization of monochrome fluorescence images were performed using Adobe Photoshop (version 7.0; Adobe, San Jose, CA). Power analysis, using data from pilot studies and other experiments using CBA/J male mice, estimated sample size at three. Most groups had a larger sample size, with three as the minimum. One-way ANOVA was used for enzyme-linked immunosorbent assay and qRT-PCR. For ABR, a two-way repeated measures ANOVA coded for treatment and day/time was used. For hair cell counts, an unpaired Student’s t-test was used. Group differences were considered significant at p < 0.05 in each case. All data presented in bar graphs are the mean ± S.E.M. from multiple determinations.

### Compliance with ethical standards

All experimental protocols were approved by Chungnam National University Institutional Animal Care and Use Committee. All animal care and use was conducted in accordance with the Guide for the Care and Use of Laboratory Animals.

## Data Availability

All data generated or analyzed during this study are included in this published article.
